# Prevalence and features of allergic bronchopulmonary aspergillosis, United States, 2016–2022

**DOI:** 10.1371/journal.pone.0317054

**Published:** 2025-01-15

**Authors:** Kaitlin Benedict, Jeremy A. W. Gold, Mitsuru Toda, Joy Hsu

**Affiliations:** 1 Mycotic Diseases Branch, Division of Foodborne, Waterborne, and Environmental Diseases, National Center for Emerging and Zoonotic Infectious Diseases, Centers for Disease Control and Prevention, Atlanta, Georgia, United States of America; 2 Asthma and Air Quality Branch, Division of Environmental Health Science and Practice, National Center for Environmental Health, Centers for Disease Control and Prevention, Atlanta, Georgia, United States of America; Dayeh University, TAIWAN

## Abstract

The epidemiology of allergic bronchopulmonary aspergillosis (ABPA) in the United States is not well-described. To estimate national ABPA prevalence among patients with asthma or cystic fibrosis, characterize ABPA testing practices, and describe ABPA clinical features, treatment, and 6-month outcomes. We used the 2016–2022 Merative™ MarketScan® Commercial/Medicare and Multi-State Medicaid Databases to identify cohorts of patients with 1) asthma, 2) cystic fibrosis (CF), and 3) ABPA. We calculated ABPA prevalence per 10,000 patients with asthma or CF, assessed diagnostic testing for ABPA among patients with severe asthma, and described features of patients with ABPA using diagnosis and procedure codes. The overall ABPA prevalence among patients with asthma was 2.8/10,000 (Commercial/Medicare) and 1.0/10,000 (Medicaid). ABPA prevalence increased with asthma severity (Commercial/Medicare: mild 1.3, moderate 9.3, severe 70.6, Medicaid: mild 0.3, moderate 2.4, severe 32.4). Among patients with CF, ABPA prevalence was 183.7/10,000 (Commercial/Medicare) and 134.6/10,000 (Medicaid). Among patients with severe asthma, 10.3% (Commercial/Medicare) and 7.4% (Medicaid) received total immunoglobulin E testing, which is recommended for ABPA diagnosis. Among all patients with ABPA (Commercial/Medicare: n = 1,564, Medicaid: n = 410), ABPA treatments included inhaled corticosteroids (>70%), systemic corticosteroids (>62%), and antifungals (>18%). Patients with ABPA and Medicaid were more likely to experience hospitalization (45.1% vs. 22.5% of patients with Commercial/Medicare insurance) and respiratory failure (18.5% vs. 10.9%). This analysis provides initial estimates of national ABPA prevalence. Further studies could identify potential barriers to ABPA testing and investigate potential factors affecting payer-related differences in ABPA burden.

## Introduction

Allergic bronchopulmonary aspergillosis (ABPA) is a pulmonary disorder caused by hypersensitivity to colonization of the airways with *Aspergillus* spp, a common environmental mold. ABPA primarily occurs among people with asthma or cystic fibrosis (CF) and can cause substantial morbidity. ABPA often presents as poorly controlled asthma, but other clinical features can include bronchiectasis and peripheral eosinophilia. Clinical complications include pulmonary hypertension and respiratory failure. Treatment includes systemic corticosteroids or antifungals. Diagnosis can be challenging and involves compatible clinical and radiological findings and laboratory results including total serum immunoglobulin E (IgE), *Aspergillus*-specific IgE and IgG, and eosinophilia. Testing for ABPA is recommended for certain patients with asthma in tertiary care settings [1, 2] and yearly screening is recommended for patients with CF [3], but testing practices in the United States have not been described using real-world data.

Globally, ABPA is estimated to affect >4.8 million people, including 1.4 million cases in the Americas [4]. This figure is based on a multi-country average ABPA prevalence of 2.5% among adults with asthma. However, recent data about the prevalence and other epidemiologic features of ABPA in the United States are lacking. According to a recent review [5], no estimates of ABPA prevalence among patients with asthma have been documented in the literature since the 1970s, which included a small study at a single specialty clinic [6]. To address this knowledge gap, we analyzed two large U.S. health insurance claims databases to estimate ABPA prevalence, describe the epidemiologic and clinical features of patients with ABPA, and describe testing patterns for ABPA among patients with severe asthma.

## Methods

We used the Merative™ MarketScan® Commercial/Medicare, and Multi-State Medicaid Databases (https://www.merative.com/documents/brief/marketscan-explainer-general). The Commercial/Medicare database contains health insurance claims data including inpatient and outpatient visits and outpatient prescriptions for >51 million employees, dependents, and retirees with employer-sponsored plans including Medicare Supplemental and Medicare Advantage plans throughout the United States during July 1, 2016 to July 1, 2022 study window. The Medicaid database contains similar information for >16 million patients across several geographically dispersed states during the same time frame. MarketScan data are fully de-identified, so this analysis was not subject to review by the Centers for Disease Control and Prevention institutional review board.

Using International Classification of Diseases, Tenth Revision, Clinical Modification (ICD-10-CM) codes (S1 Table), we identified cohorts of patients diagnosed with either 1) asthma, stratified by severity, 2) cystic fibrosis (CF), or 3) ABPA during the study window who had continuous insurance enrollment in the 180 days before and after their first diagnosis code for the condition of interest (index date). Patients for whom the condition of interest was listed on a laboratory or imaging claim alone were excluded from the cohort to decrease likelihood of misclassification.

For the asthma and CF cohorts, we calculated ABPA prevalence per 10,000 patients, stratified by demographic characteristics and insurance type. Using both databases, we also assessed ordering for ABPA-related diagnostic testing among patients with severe asthma or cystic fibrosis using Current Procedural Terminology (CPT) codes documented in the 60 days before to 60 days after the index date. For the ABPA cohort, we examined patient demographic characteristics, other selected concomitant diagnoses using ICD-10-CM codes, diagnostic testing, outpatient medications, and outcomes. Geographic data (Census region and rural classification) are unavailable in the Medicaid database, and race/ethnicity data are unavailable in the Commercial/Medicare database. The Commercial/Medicare and Medicaid databases were not combined or compared with statistical testing because of the different sampling methodology.

## Results

### ABPA prevalence

The overall prevalence of ABPA among patients with any asthma diagnosis was 2.8/10,000 patients (n = 715/2,527,072) in the Commercial/Medicare database (Table 1) and 1.0/10,000 (n = 163/1,631,142) in the Medicaid database (Table 2). ABPA prevalence increased with increasing asthma severity (Commercial/Medicare: mild 1.3, moderate 9.3, severe 70.6, Medicaid: mild 0.3, moderate 2.4, severe 32.4). ABPA prevalence was typically highest among adults ≥45 years and higher among males vs. females, with the most pronounced difference among patients with severe asthma in the Commercial/Medicare database (males: 82.9, females: 62.1). In the Commercial/Medicare database, ABPA prevalence among patients with moderate asthma was highest in the West (14.1), and ABPA prevalence among patients with severe asthma was highest in the Midwest (89.9). ABPA prevalence was similar in rural vs. non-rural areas among patients with mild and moderate asthma but was higher in rural vs. non-rural areas (90.0 vs. 61.4) among patients with severe asthma. In the Medicaid database, ABPA prevalence among patients with severe asthma was highest among non-Hispanic Black patients (36.5, vs. 26.3 among non-Hispanic White patients and 22.1 among Hispanic/Latino patients).

**Table 1:** ABPA rates^a^ per 10,000 patients with Commercial/Medicare insurance and asthma or cystic fibrosis.

	Asthma					Cystic fibrosis
	Any	Mild	Moderate	Severe	Unspecified severity	
Total	715/2527072 (2.8)	113/860619 (1.3)	142/152146 (9.3)	112/15856 (70.6)	385/1538311 (2.5)	97/5279 (183.7)
Sex						
Male	319/1057050 (3.0)	48/385218 (1.2)	68/64328 (10.6)	54/6511 (82.9)	169/619305 (2.7)	51/2483 (205.4)
Female	396/1470022 (2.7)	65/475401 (1.4)	74/87818 (8.4)	58/9345 (62.1)	216/919006 (2.4)	46/2796 (164.5)
Age group in years						
<18	37/676654 (0.5)	4/304089 (0.1)	6/32743 (1.8)	5/2131 (23.5)	24/356641 (0.7)	31/1769 (175.2)
18 to 44	159/859702 (1.8)	23/275992 (0.8)	20/23393 (8.5)	23/4323 (53.2)	101/542846 (1.9)	50/2189 (228.4)
45 to 64	390/809270 (4.8)	31/120946 (2.6)	74/58768 (12.6)	68/7320 (92.9)	198/515725 (3.8)	15/1059 (141.6)
≥65	129/181446 (7.1)	14/44467 (3.1)	42/13849 (30.3)	16/2082 (76.8)	62/123099 (5.0)	1/262 (38.2)
US census region^b^						
Northeast	117/473609 (2.5)	21/171971 (1.2)	20/25350 (7.9)	16/2396 (66.8)	63/281491 (2.2)	14/1087 (128.8)
Midwest	147/530286 (2.8)	22/118382 (1.9)	29/34690 (8.4)	32/3561 (89.9)	83/312966 (2.7)	24/1124 (213.5)
South	227/982408 (2.3)	39/320896 (1.2)	42/58657 (7.2)	36/6249 (57.6)	119/612137 (1.9)	29/2025 (143.2)
West	160/408545 (3.9)	21/140417 (1.5)	34/24079 (14.1)	17/2346 (72.5)	90/247215 (3.6)	24/746 (321.7)
Unknown	64/132224 (4.8)	9/38953 (2.3)	17/9370 (18.1)	11/1304 (84.4)	30/84052 (3.6)	6/297 (202.0)
Rural classification						
Non-rural	488/1799133 (2.7)	84/628246 (1.3)	91/106824 (8.5)	66/10748 (61.4)	270/1081982 (2.5)	66/3715 (177.7)
Rural	62/252648 (2.5)	8/74182 (1.1)	13/15605 (8.3)	15/1667 (90.0)	29/164648 (1.8)	9/586 (153.6)
Unknown	165/475291 (3.5)	21/158191 (1.3)	38/29717 (12.8)	31/3441 (90.1)	86/291681 (2.9)	22/978 (224.9)

^a^ table shows numerator/denominator (rate per 10,000)

^b^ of primary beneficiary’s residence

**Table 2:** ABPA rates^a^ per 10,000 patients with Medicaid and asthma or cystic fibrosis.

	Asthma					Cystic fibrosis
	Any	Mild	Moderate	Severe	Unspecified severity	
Total	163/1631142 (1.0)	15/554302 (0.3)	22/90034 (2.4)	28/8636 (32.4)	111/1028613 (1.1)	54/4013 (134.6)
Sex						
Male	89/738416 (1.2)	10/279874 (0.4)	11/43447 (2.5)	13/3949 (32.9)	64/437852 (1.5)	31/1887 (164.3)
Female	74/892726 (0.8)	5/274428 (0.2)	11/46587 (2.4)	15/4687 (32.0)	47/590761 (0.8)	23/2126 (108.2)
Age group in years						
<18	54/1022654 (0.5)	9/429497 (0.2)	13/59037 (2.2)	10/4438 (22.5)	32/571571 (0.6)	27/2363 (114.3)
18 to 44	62/423842 (1.5)	4/89543 (0.4)	6/18477 (3.2)	7/2214 (31.6)	46/319879 (1.4)	26/1356 (191.7)
45 to 64	42/175733 (2.4)	2/33596 (0.6)	2/11925 (1.7)	11/1892 (58.1)	29/130509 (2.2)	1/156 (64.1)
≥65	5/8913 (5.6)	0/1666 (0.0)	1/595 (16.8)	0/92 (0.0)	4/6654 (6.0)	0/24 (0.0)
Race/ethnicity						
Black, non-Hispanic	56/589547 (0.9)	6/213220 (0.3)	8/33083 (2.4)	13/3563 (36.5)	38/362609 (1.0)	4/623 (64.2)
Hispanic or Latino	12/107817 (1.1)	2/42205 (0.5)	3/5885 (5.1)	1/452 (22.1)	8/63209 (1.3)	4/177 (226.0)
Other race, non-Hispanic	1/64267 (0.2)	0/25109 (0.0)	0/3556 (0.0)	0/321 (0.0)	1/37282 (0.3)	1/98 (102.0)
White, non-Hispanic	68/726349 (0.9)	3/227164 (0.1)	8/38786 (2.1)	9/3425 (26.3)	49/474081 (1.0)	36/2493 (144.4)
Unknown	26/143162 (1.8)	4/46604 (0.9)	3/8724 (3.4)	5/875 (57.1)	15/91432 (1.6)	9/622 (144.7)

^a^ table shows numerator/denominator (rate per 10,000)

Among patients with cystic fibrosis, the overall prevalence of ABPA was 183.7/10,000 patients (n = 97/5,279) in the Commercial/Medicare database and 134.6/10,000 in the Medicaid database (n = 54/4,013). ABPA prevalence among patients with CF was highest among adults ages 18–44 years (Commercial/Medicare: 228.4, Medicaid: 191.7) and males (Commercial/Medicare: 205.4 vs. 164.5 in females, Medicaid: 164.3 vs. 108.2 in females). In the Commercial/Medicare database, ABPA prevalence among patients with CF was highest in the West (321.7).

### ABPA-related testing among patients with severe asthma or cystic fibrosis

Among patients with severe asthma (Commercial/Medicare: n = 15,856, Medicaid: n = 8,636),

10.3% and 7.4%, respectively, received total serum IgE testing, 6.5% and 5.6% received allergen specific IgE testing, and 1.0% and 0.5% received testing for *Aspergillus* precipitating antibodies (Table 3).

**Table 3:** ABPA-related diagnostic testing^a^ among patients with severe asthma or cystic fibrosis, by health insurance type.

	Severe asthma		Cystic fibrosis	
	Commercial/Medicare	Medicaid	Commercial/Medicare	Medicaid
	n = 15,856 (%)	n = 8,636 (%)	n = 5,279 (%)	n = 4,013 (%)
*Aspergillus* precipitating antibodies	152 (1.0%)	42 (0.5%)	41 (0.8%)	9 (0.2%)
Fungal culture	171 (1.1%)	68 (0.8%)	583 (11.0%)	241 (6.0%)
Microscopy	492 (3.1%)	234 (2.7%)	1,255 (23.8%)	726 (18.1%)
*Aspergillus *galactomannan antigen detection	19 (0.1%)	8 (0.1%)	36 (0.7%)	12 (0.3%)
Serum immunoglobulin E (IgE)	1,631 (10.3%)	642 (7.4%)	802 (15.2%)	448 (11.2%)
Allergen-specific IgE	1,025 (6.5%)	484 (5.6%)	142 (2.7%)	66 (1.6%)
Chest CT	1,328 (8.4%)	426 (4.9%)	499 (9.5%)	272 (6.8%)
Spirometry	7,013 (44.2%)	3,140 (36.4%)	2,345 (44.4%)	1,423 (35.5%)
Bronchoscopy	200 (1.3%)	110 (1.3%)	190 (3.6%)	146 (3.6%)

ABPA: allergic bronchopulmonary aspergillosis; CT: computed tomography; IgE: immunoglobulin E

^a^ In the 60 days before to 60 days after the first severe asthma or CF diagnosis in the study window

Among patients with cystic fibrosis (Commercial/Medicare: n = 5,279, Medicaid: n = 4,013), 15.2% and 11.2%, respectively, received total serum IgE testing, 2.7% and 1.6% received allergen specific IgE testing, and 0.8% and 0.2% received testing for *Aspergillus* precipitating antibodies.

### Features of patients with ABPA

Among all patients with ABPA and commercial/Medicare insurance (n = 1,564), the most common specified provider types visited on the index date were pulmonologists (36.6%) and acute care hospitals (35.5%). In contrast, among patients with ABPA and Medicaid insurance (n = 410), acute care hospitals (51.0%) were the most common specified provider type on the index date (Table 4). ABPA index dates most frequently occurred in the summer (Commercial/Medicare: 32.7%, Medicaid: 30.0%).

**Table 4:** Demographic characteristics, clinical features, treatments, and outcomes among patients with ABPA, by health insurance type.

	Commercial/Medicare		Medicaid	
Characteristic	n = 1,564	%	n = 410	%
Sex				
Male	708	45.3%	203	49.5%
Female	856	54.7%	207	50.5%
Age group in years				
<18	78	5.0%	123	30.0%
18 to 44	342	21.9%	152	37.1%
45 to 64	841	53.8%	124	30.2%
≥65	303	19.4%	11	2.7%
US census region of primary beneficiary’s residence				
Northeast	220	14.1%	n/a	n/a
Midwest	335	21.4%	n/a	n/a
South	521	33.3%	n/a	n/a
West	359	23.0%	n/a	n/a
Unknown	129	8.2%	n/a	n/a
Urban/rural classification				
Non-rural	1,059	67.7%	n/a	n/a
Rural	149	9.5%	n/a	n/a
Unknown	356	22.8%	n/a	n/a
Race/ethnicity				
Black, non-Hispanic	n/a	n/a	133	32.4%
Hispanic or Latino	n/a	n/a	30	7.3%
Other race, non-Hispanic	n/a	n/a	12	2.9%
White, non-Hispanic	n/a	n/a	184	44.9%
Unknown	n/a	n/a	51	12.4%
Season of ABPA diagnosis				
Winter	344	22.0%	95	23.2%
Spring	303	19.4%	86	21.0%
Summer	511	32.7%	123	30.0%
Fall	406	26.0%	106	25.9%
Provider type(s) on day of ABPA diagnosis (not mutually exclusive)				
Acute care hospital	556	35.5%	209	51.0%
Allergy/immunology	152	9.7%	6	1.5%
Family practice or internal medicine	328	21.0%	25	6.1%
Laboratory	123	7.9%	14	3.4%
Other	510	32.6%	239	58.3%
Pediatrician	69	4.4%	31	7.6%
Pulmonary disease	573	36.6%	21	5.1%
Radiology	149	9.5%	12	2.9%
Unknown	33	2.1%	93	22.7%
Conditions in the 180 days before to 180 days after ABPA diagnosis				
Acute sinusitis	286	18.3%	47	11.5%
Acute upper respiratory infection	218	13.9%	83	20.2%
Allergic rhinitis	726	46.4%	190	46.3%
Anxiety disorder	259	16.6%	106	25.9%
Asthma	1,175	75.1%	309	75.4%
Mild	185	11.8%	35	8.5%
Moderate	274	17.5%	57	13.9%
Severe	162	10.4%	52	12.7%
Unspecified severity	564	36.1%	175	42.7%
Bronchiectasis	649	41.5%	145	35.4%
Chronic obstructive pulmonary disease (COPD)	465	29.7%	135	32.9%
Chronic sinusitis	445	28.5%	98	23.9%
Cystic fibrosis	185	11.8%	122	29.8%
Depression	212	13.6%	101	24.6%
Diabetes	286	18.3%	89	21.7%
Eosinophilia	141	9.0%	43	10.5%
Functional disorders of polymorphonuclear neutrophils	10	0.6%	2	0.5%
Gastroesophageal reflux disease (GERD)	534	34.1%	172	42.0%
Hyper-IgE syndrome	30	1.9%	7	1.7%
Hypertension	609	38.9%	137	33.4%
Hypothyroidism	246	15.7%	23	5.6%
Liver disease	134	8.6%	53	12.9%
Lung transplant	12	0.8%	1	0.2%
Nontuberculous mycobacteria infection	83	5.3%	17	4.1%
Overweight and obesity	242	15.5%	85	20.7%
Pneumonia	540	34.5%	174	42.4%
Smoking (current or past)	246	15.7%	129	31.5%
Vitamin D deficiency	267	17.1%	85	20.7%
Diagnostic testing in the 60 days before to 60 days after ABPA diagnosis				
*Aspergillus* precipitating antibodies	271	17.3%	34	8.3%
Fungal culture	262	16.8%	53	12.9%
Microscopy	371	23.7%	91	22.2%
*Aspergillus *galactomannan antigen detection	98	6.3%	14	3.4%
Serum IgE	633	40.5%	142	34.6%
Allergen-specific IgE	349	22.3%	88	21.5%
Chest CT	624	39.9%	149	36.3%
Spirometry	786	50.3%	208	50.7%
Bronchoscopy	230	14.7%	44	10.7%
Outpatient medications in the 7 days before to 180 days after ABPA diagnosis				
Inhaled corticosteroids	1106	70.7%	296	72.2%
Systemic corticosteroids	969	62.0%	266	64.9%
≤7 days supply	110	11.4%	44	16.5%
8–14 days supply	100	10.3%	26	9.8%
15–30 days supply	168	17.3%	35	13.2%
≥31 days supply	591	61.0%	161	60.5%
Interleukin (IL)-5 agents	37	2.4%	10	2.4%
Mepolizumab	23	1.5%	7	1.7%
Benralizumab	15	1.0%	3	0.7%
Additional monoclonal antibodies	129	8.2%	31	7.6%
Omalizumab	100	6.4%	22	5.4%
Dupilumab	29	1.9%	10	2.4%
Antifungals	397	25.4%	77	18.8%
Itraconazole	210	13.4%	36	8.8%
Voriconazole	182	11.6%	39	9.5%
Posaconazole	27	1.7%	7	1.7%
Both systemic corticosteroids and antifungals	313	20.0%	64	15.6%
Outcomes on or in the 180 days after ABPA diagnosis				
Hospitalized	352	22.5%	185	45.1%
Median days hospitalized (IQR)	5	(3–8)	6	(3–12)
Bronchiectasis	544	34.8%	115	28.0%
Respiratory failure	171	10.9%	76	18.5%
Invasive aspergillosis	41	2.6%	7	1.7%
Median number of healthcare visits in the 180 days after ABPA diagnosis (IQR)	13	(6–23)	13	(6–26)
>1 healthcare visit for ABPA	930	59.5%	232	56.6%

ABPA: allergic bronchopulmonary aspergillosis; CT: computed tomography; IgE: immunoglobulin E; IQR: interquartile range.

The most frequent associated diagnoses were asthma (75.1%), allergic rhinitis (46.4%), bronchiectasis (41.5%), and hypertension (38.9%) among patients with ABPA and commercial/Medicare insurance. Among patients with Medicaid, the most common associated diagnoses were asthma (75.4%), allergic rhinitis (46.3%), pneumonia (42.4%), and gastrointestinal reflux disease (42.0%). Cystic fibrosis occurred in 11.7% of patients with commercial/Medicare insurance and 29.8% of patients with Medicaid; 18.5% and 12.7% of patients with commercial/Medicare insurance and Medicaid, respectively, did not have diagnosis codes for either CF or asthma.

Diagnostic testing included *Aspergillus* precipitating antibodies (Commercial/Medicare: 17.3%, Medicaid: 8.3%), total serum IgE (Commercial/Medicare: 40.5%, Medicaid: 30.6%), and allergen specific IgE (Commercial/Medicare: 22.3%, Medicaid: 21.5%). Most patients were prescribed inhaled (Commercial/Medicare: 70.7%, Medicaid: 72.2%) or systemic (Commercial/Medicare: 62.0%, Medicaid: 64.9%) corticosteroids. The median days’ supply for systemic steroids was 56 (interquartile range [IQR] 18–128) days for patients with commercial/Medicare insurance and 49 (IQR 14–117) days for patients with Medicaid. Antifungals, most commonly itraconazole, were prescribed for 25.4% of patients with commercial/Medicare insurance and 18.8% of patients with Medicaid; 20.0% of patients with commercial/Medicare insurance and 15.6% of patients with Medicaid received both antifungals and systemic corticosteroids. The median days’ supply for itraconazole was 90 days (IQR: 30–120) for patients with commercial/Medicare insurance and 60 days (IQR 30–133.5) for patients with Medicaid. Approximately 2% were prescribed interleukin (IL)-5 agents and an additional 8% of patients in each cohort were prescribed other monoclonal antibodies.

On or in the 180 days after the index date, 22.5% of patients with commercial/Medicare insurance and 45.1% of patients with Medicaid were hospitalized, 10.9% of patients with commercial/Medicare insurance and 18.5% of patients with Medicaid experienced respiratory failure, approximately 2% of patients in each cohort developed invasive aspergillosis, and patients from both cohorts had a median of 13 additional healthcare visits. Most (Commercial/Medicare: 59.5%, Medicaid: 56.6%) patients had more than one additional healthcare visit with an ABPA diagnosis code; among those, the median time between the index date and the last ABPA visit was 119 days (IQR 59–55) for patients with commercial/Medicare insurance and 115 days (IQR 53–154) for patients with Medicaid.

## Discussion

This analysis of two large health insurance claims databases provides initial estimates of the prevalence of ABPA among patients with asthma or CF and offers preliminary insight into nationwide testing and treatment patterns for ABPA in the United States. ABPA prevalence was highest among patients with CF and among those with severe asthma, and we observed differences in prevalence by demographic characteristics and insurance type. Testing for ABPA appeared to be infrequent, suggesting that additional studies could help better understand testing practices and identify potential barriers to testing for ABPA.

Globally, the prevalence of ABPA among adult patients with asthma is estimated to be approximately 2.5 to 11% based on studies mainly from tertiary care centers [4, 5, 7]. In our analysis, the prevalence of ABPA was substantially lower, even specifically among patients with severe asthma (0.3%–0.7%). Our finding of increasing ABPA prevalence with increasing asthma severity contrasts with a recent study from India and may be related to higher ABPA testing rates among patients with severe asthma compared to those with mild asthma, which we did not evaluate [8]. The differences between our findings and previous studies are likely because of differences in study design and patient population, as our study included children and patients across all healthcare settings. Studies of ABPA prevalence worldwide also show that the reported prevalence varies greatly by country [4, 5, 7]. Our study suggests that geographic variation also occurs within the United States, with higher ABPA prevalence in the West and Midwest among patients with commercial health insurance. This finding is consistent with the geographic distribution of hospitalizations involving invasive aspergillosis [9] and with aspergillosis-related deaths [10]; however, the reasons for this pattern are unclear. Further investigation could improve understanding, particularly because the risk for developing ABPA is believed to be due to genetic rather than environmental factors [11, 12]. The higher rates of ABPA we observed among patients with severe asthma in rural areas is also intriguing because both asthma and invasive aspergillosis are more prevalent in urban areas [13, 14]. This association could be related to our finding that ABPA was most common in middle-age and older adults, who comprise a larger proportion of the population in rural areas [15]. Despite asthma being more common among female patients [16], we found that ABPA was more prevalent among male patients, similar to invasive aspergillosis and many other fungal infections [13]. By race and ethnicity, ABPA prevalence among patients with Medicaid appeared to mirror asthma prevalence in the general population [16] with the highest rates among non-Hispanic Black persons, followed by non-Hispanic White and Hispanic persons.

The slightly higher ABPA prevalence among patients in the Commercial/Medicare dataset compared with the Medicaid dataset is consistent with a nationwide study showing that rates of hospitalizations involving non-invasive aspergillosis were 11.1 per 100,000 population among patients with private insurance, 13.6 among those with Medicare, and 11.7 among those with Medicaid [13]. Asthma prevalence is higher among persons with lower socioeconomic status [16], so our findings suggest that the difference in ABPA by insurance type may be due to factors besides asthma prevalence. The difference could be related to the age distribution of the two study populations, since the Medicaid population is younger, and ABPA appears to more frequently affect adults. Another explanation involves differences in healthcare seeking behavior or access (including access to specialists), as supported by the variation in provider type among patients with commercial/Medicare insurance vs. Medicaid. The differences could also be partly attributable to frequency of testing for ABPA among patients with severe asthma, which were slightly lower among patients with Medicaid. Notably, certain unfavorable outcomes (e.g., respiratory failure) were more common among patients with ABPA and Medicaid compared with those with commercial/Medicare insurance, which could be related to other factors influencing overall health status (e.g., smoking) or access to (or delays in) receiving medical care.

Overall, specific diagnostic testing for ABPA was uncommonly documented among patients with severe asthma or CF. Our study was designed to identify testing for ABPA within the 2 months before or after a patient’s first diagnosis of severe asthma or CF in the study window, and it is possible that more testing could be identified with a longer time frame or with a focus on incident ABPA visits. Guidelines from the Infectious Diseases Society of America (IDSA) recommend testing for ABPA among patients with asthma in tertiary care settings and yearly screening for patients with asthma or CF [2]. Similarly, the International Society for Human and Animal Mycology (ISHAM) recommends *Aspergillus* IgE testing for adults newly diagnosed with asthma in tertiary care settings [1]. A reassuring finding is that a small proportion of patients with ABPA diagnosis codes were tested with fungal culture (17%) or *Aspergillus* galactomannan (<7%). Sputum culture is not explicitly recommended for diagnosing ABPA because of the low sensitivity and specificity but might be useful for monitoring response to therapy and understanding azole resistance [1]. *Aspergillus* galactomannan testing is also not recommended for diagnosing ABPA [1], but some patients with ABPA may have received this test for invasive pulmonary aspergillosis. Surveys of providers who most frequently see patients with severe asthma or CF, such as pulmonologists and healthcare providers based at acute care hospitals, might help better understand testing practices and identify possible gaps in and barriers to testing for ABPA. Prompt diagnosis of ABPA is important for improving outcomes because delayed treatment can lead to poor outcomes such as bronchiectasis and worsening lung function [17].

Most (>62%) patients with ABPA in this analysis received systemic corticosteroids, which are the mainstay of treatment for ABPA [1]. The median duration of steroid treatment in our analysis (<2 months) was shorter than the ISHAM-recommended 4-month tapered treatment course [1], although our study might underestimate total treatment duration because the databases do not contain information about medications administered in the inpatient setting. Oral itraconazole, received by 19–25% of patients with ABPA in this analysis, is the recommended alternative to corticosteroids; again, the prescribed days supply was shorter than the recommended 4 months. Over 15% of patients with ABPA received both systemic corticosteroids and antifungals at some point during the follow-up window. Concurrent use of steroids and antifungals is not recommended except for certain patients, such as those with eosinophilia, extensive bronchiectasis, or recurrent ABPA exacerbation [1]. We did not assess medication-related adverse effects, which can be substantial with corticosteroids and antifungals [17], or patient adherence. We were also unable to evaluate medication dosages. Biologic treatments were infrequently used in this analysis (<9%); these are promising for difficult-to-control ABPA (for example, ABPA requiring repeated courses of corticosteroids or antifungals to control ABPA activity and symptoms) but have not been studied with large-scale trials [17]. Additional, more detailed analyses could help to identify patient- and provider-related factors associated with treatment type and association of treatment type with outcomes.

The median duration of nearly 4 months between patients’ first and last ABPA-related visit and the substantial number of subsequent healthcare visits in the follow up period suggests that ABPA poses a considerable burden to patients. A slightly higher proportion of patients had their first ABPA-related visit during the summer, similar to findings from a study of invasive aspergillosis among patients who received hematopoietic stem cell transplants [18]. A better understanding of seasonal and climate factors influencing the risk for illness caused by *Aspergillus* could allow for recognition of higher-risk time periods. ABPA is believed to be uncommon in patients without either asthma or cystic fibrosis [19]; our results suggest that it may be more common than previously appreciated, or more likely, that we were unable to capture underlying conditions completely with ICD-10-CM codes recorded in the 180 days before the index date [20].

Our study is subject to several limitations, particularly underrepresentation and selection bias. Although the Medicaid data sample is drawn from geographically dispersed states, information about which states were included was not available. Therefore, geographic variation in Medicaid use may mean that our results do not necessarily reflect the entire Medicaid population. The Commercial/Medicare data are broadly representative of commercially insured persons throughout the United States, but our study does not represent people with other types of health insurance or people without health insurance.

Another major study limitation is under-detection inherently present in medical claims data, which may not capture all diagnoses or procedures. Misclassification of recorded diagnoses and procedures is another concern with claims data. Claims data have been widely used to study asthma, with a variety of case-finding algorithms. A systematic literature review of the validity of claims-based algorithms showed that use of ≥1 diagnostic code for asthma over a one-year period is generally a valid method to identify cases [20]. We chose to use this broad definition of asthma to obtain larger denominators of patients with asthma and calculate more conservative estimates of ABPA. Future studies can refine these estimates by using more restrictive or standardized definitions of asthma, such as the well-established Healthcare Effectiveness Data and Information Set persistent asthma criteria. Classifying asthma severity is challenging with claims data, and no clear ideal method exists to do so [21, 22]. We may have underestimated the number of patients with severe asthma if those patients received ICD-10-CM codes for unspecified asthma, which comprised a sizeable proportion of all patients with any asthma diagnosis. We are unaware of any studies demonstrating the ability of the ICD-10-CM code to accurately identify ABPA. For invasive mold infections, use of ICD-10-CM codes for case identification might miss 25% to 50% of cases [23]. Differences in ABPA coding may occur by provider type, which may influence our analysis given the variety of provider types visited by patients with ABPA. Furthermore, we were unable to identify ABPA exacerbations or conditions such as *Aspergillus* sensitization or chronic pulmonary aspergillosis using ICD-10-CM codes, and the datasets do not include information about laboratory, imaging, or spirometry test results or mortality. Lastly, the low rate of testing for ABPA among patients with severe asthma suggest that our results might under-estimate the true prevalence of ABPA if it is not being adequately tested for and diagnosed.

This study of real-world health insurance claims data provides an estimate of the prevalence of ABPA in the United States as well as insight into ABPA testing and treatment patterns. These results can be used as a benchmark for future studies or public health surveillance to better define the burden of ABPA in the United States and to identify opportunities for improved strategies for ABPA prevention and treatment.

## Supporting information

S1 Table International Classification of Diseases, Tenth Revision, Clinical Modification (ICD-10-CM) and Current Procedural Terminology (CPT) codes used to identify diagnoses and procedures of interest. (DOCX)
