# Supplementary material for: Prevalence and features of allergic bronchopulmonary aspergillosis, United States, 2016–2022
Source: PLoS One. 2025 Jan 15;20(1):e0317054. doi: 10.1371/journal.pone.0317054 (PMC11734977; doi:10.1371/journal.pone.0317054)
Supplement: S1 Table — (DOCX) [file pone.0317054.s001.docx]

| **Description** | **ICD-10-CM code(s)** |
| --- | --- |
| Acute sinusitis | J01 |
| Acute upper respiratory infection | J06 |
| Allergic bronchopulmonary aspergillosis (ABPA) | B44.81 |
| Allergic rhinitis | J30 |
| Anxiety disorder | F40-F41 |
| Asthma | J45 |
| Mild | J45.2, J45.3 |
| Moderate | J45.4 |
| Severe | J45.5 |
| Unspecified severity | J45.9 |
| Bronchiectasis | J47 |
| Chronic obstructive pulmonary disease (COPD) | J43-J44 |
| Chronic sinusitis | J32 |
| Cystic fibrosis | E84 |
| Depression | F32-F33 |
| Diabetes | E10-E13 |
| Eosinophilia | D72.1 |
| Functional disorders of polymorphonuclear neutrophils | D71 |
| Gastroesophageal reflux disease (GERD) | K21 |
| Hyper-IgE syndrome | D82.4 |
| Hypertension | I10 |
| Hypothyroidism | E03 |
| Invasive aspergillosis | B44.0, B44.7 |
| Liver disease | K70-K77 |
| Lung transplant | Z94.2, Z94.3, T86.3, T86.81 |
| Overweight and obesity | E66 |
| Nontuberculous mycobacteria infection | A31 |
| Pneumonia | J12-J18 |
| Respiratory failure | J96 |
| Smoking (current or past) | F17, Z87.891 |
| Vitamin D deficiency | E55 |
| **Description** | **CPT code(s)** |
| *Aspergillus*galactomannan antigen detection | 87305 |
| *Aspergillus* precipitating antibodies | 86606 |
| Fungal culture | 87101, 87102, 87013, 87106, 87107 |
| Microscopy | 87205, 87206, 87208, 87210, 87211, 87220 |
| Serum immunoglobulin E (IgE) | 82785 |
| Quantitative or semi-quantitative in vitro allergen specific IgE testing | 86003 |
| Chest CT | 71250, 71260, 71270 |
| Spirometry | 3023F, 3025F, 3027F, 94010, 94011, 94012, 94014, 94015, 94016, 94060, 94070, 94617, G8924, G8925 |
| Bronchoscopy | 31620, 31622, 31623, 31624, 31625, 31626, 31627, 31628, 31629, 31630, 31631, 31632, 31633, 31634, 31635, 31636, 31637, 31638, 31640, 31641, 31643, 31645, 31646, 31647, 31648, 31649, 31651, 31652, 31653, 31654, 31656, 31659, 31660, 31661 |
